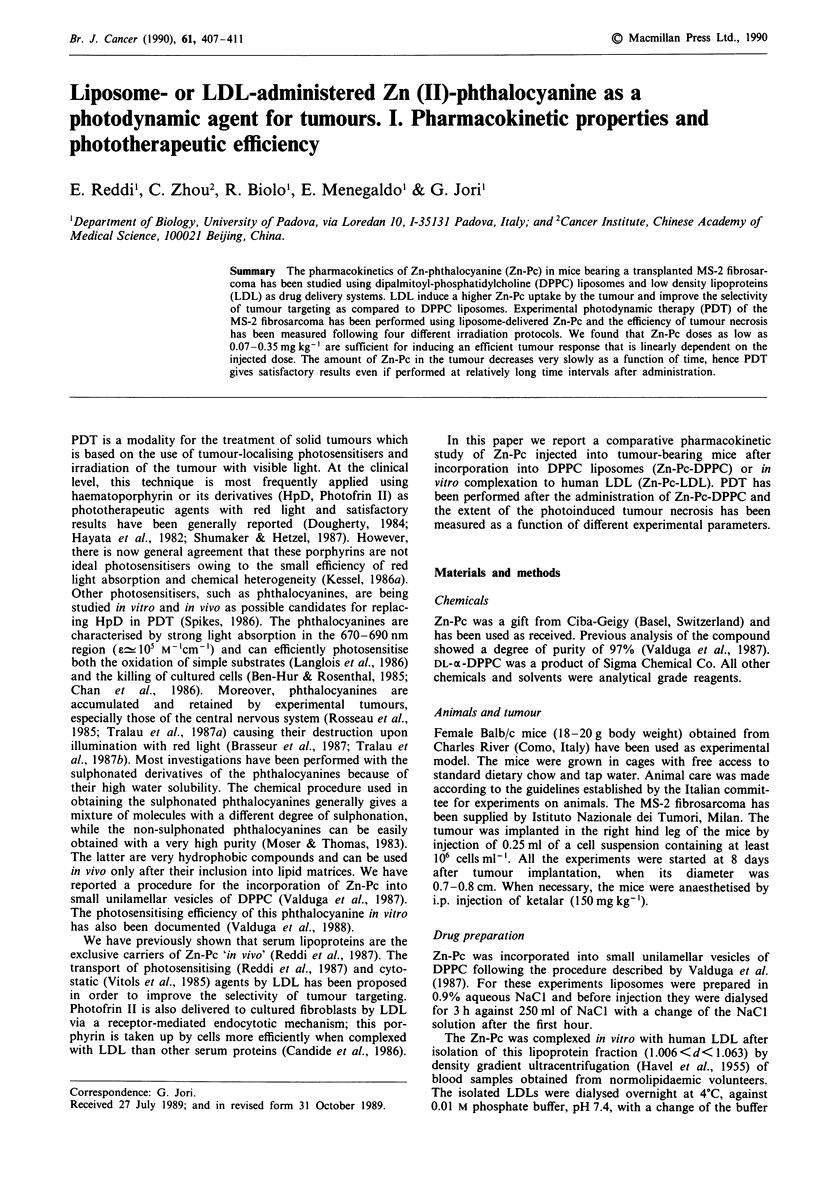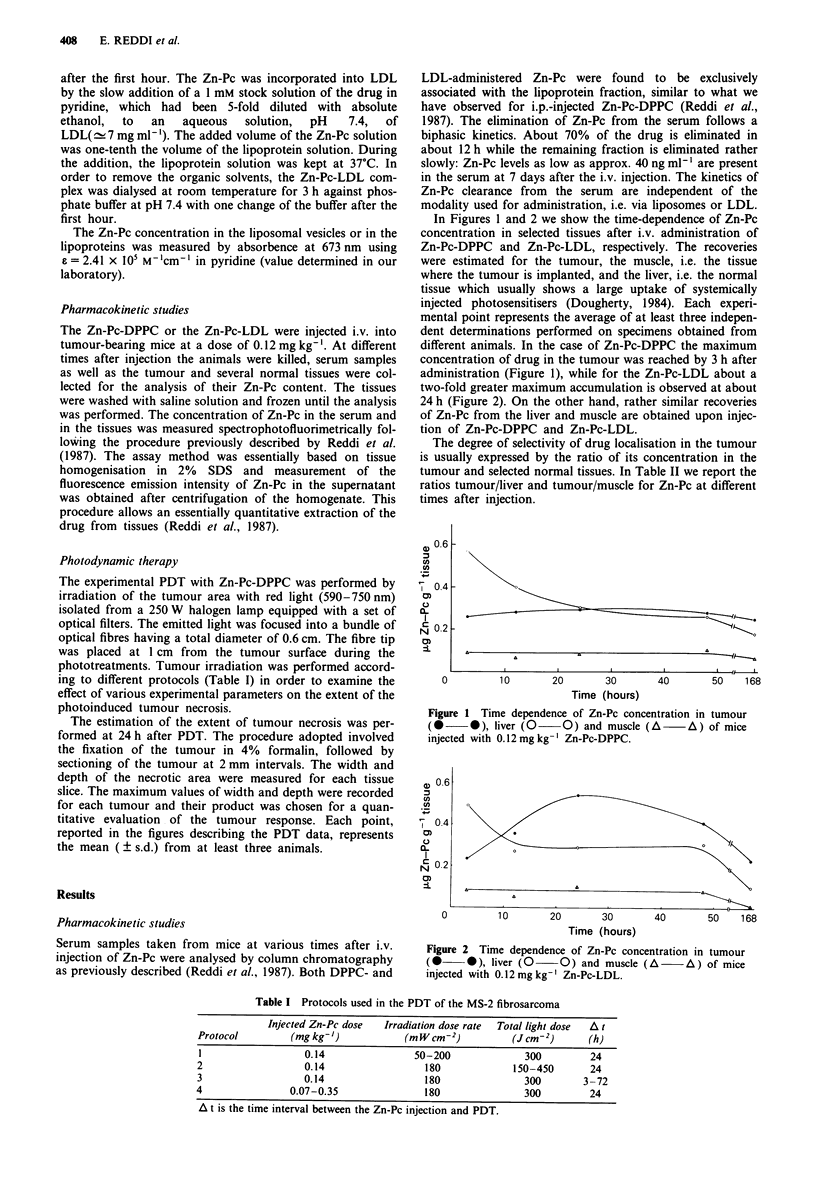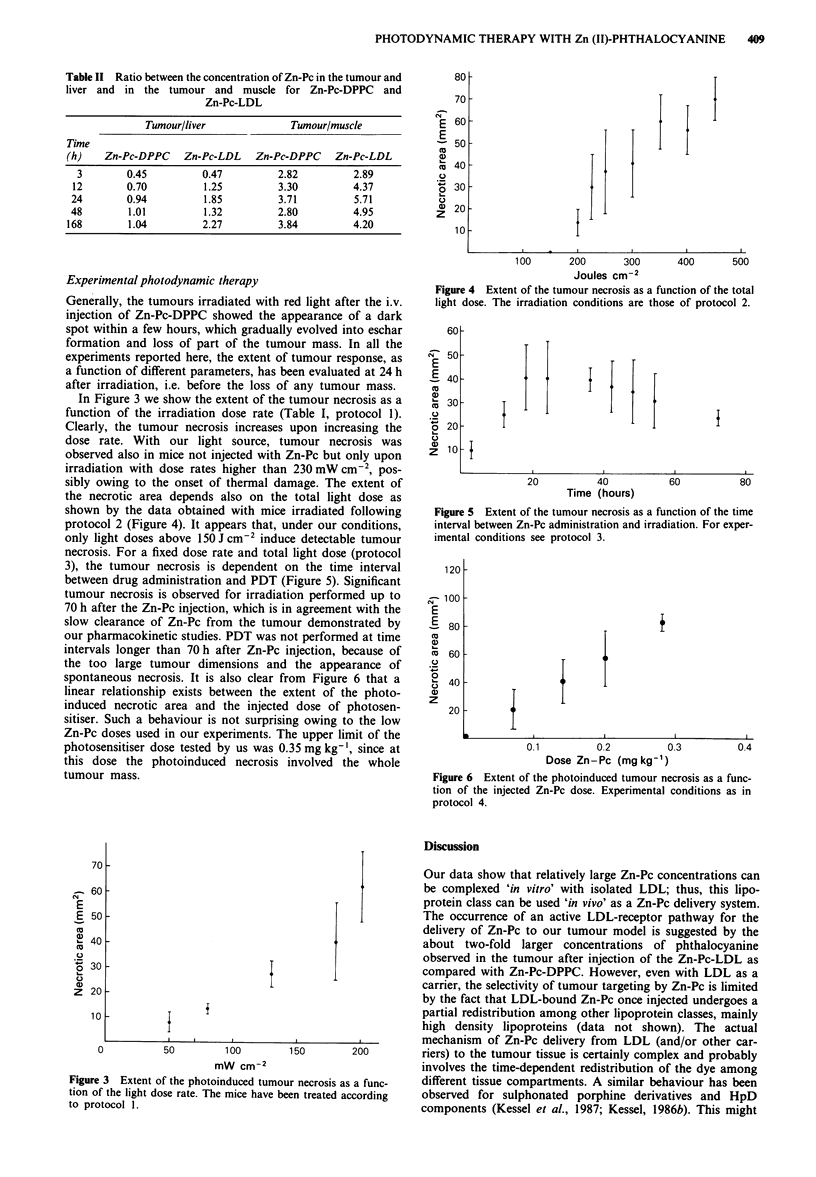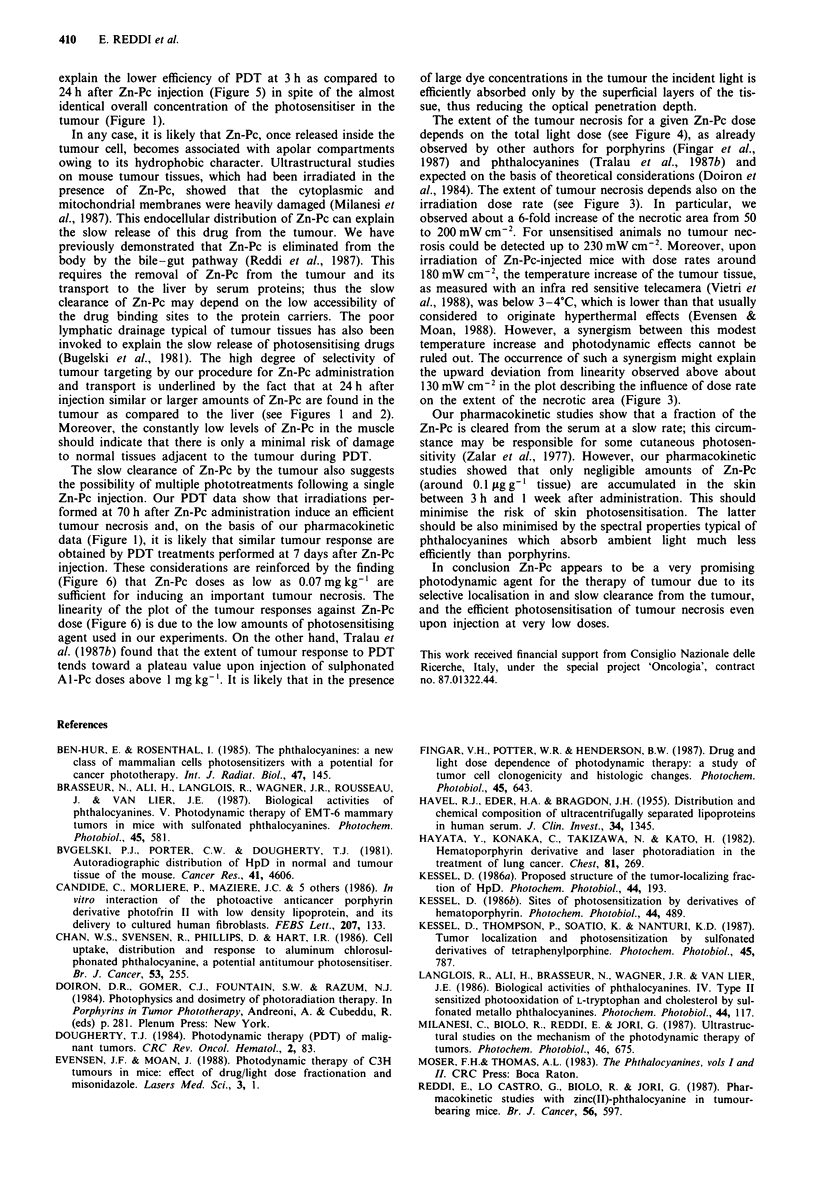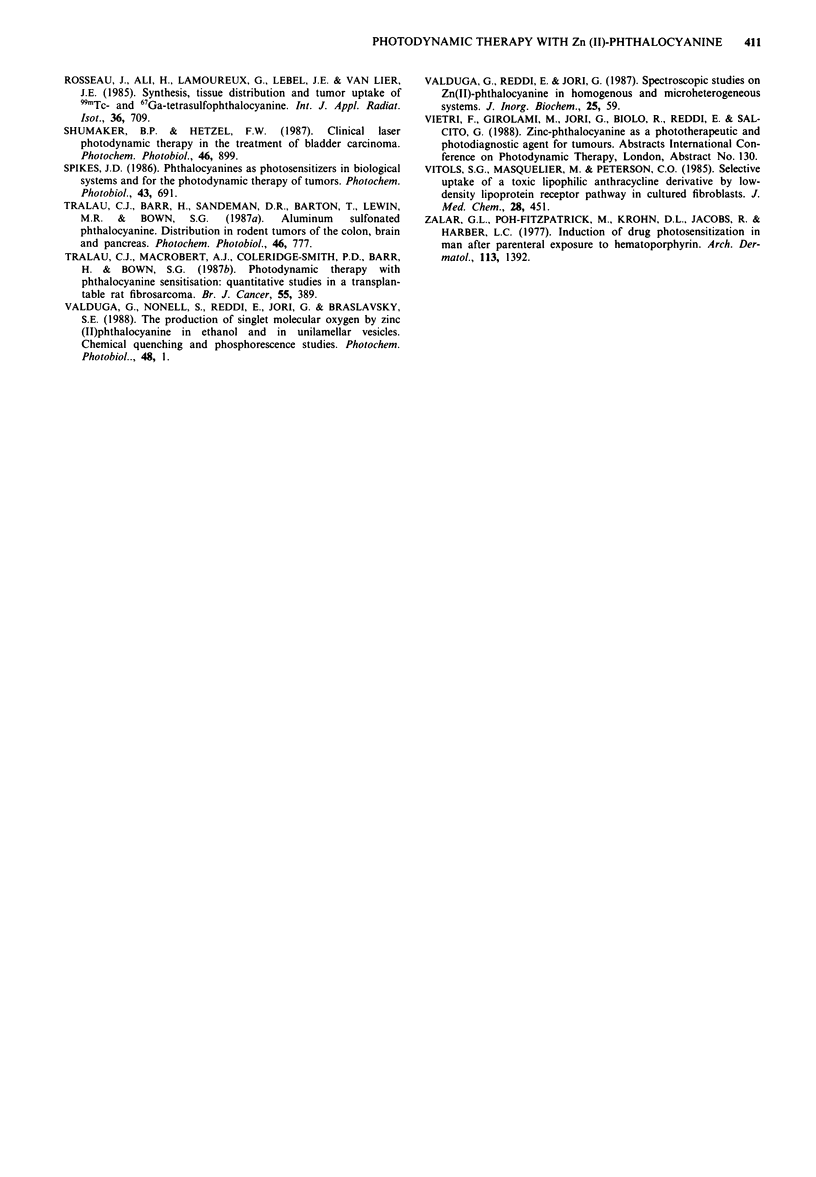# Liposome- or LDL-administered Zn (II)-phthalocyanine as a photodynamic agent for tumours. I. Pharmacokinetic properties and phototherapeutic efficiency.

**DOI:** 10.1038/bjc.1990.89

**Published:** 1990-03

**Authors:** E. Reddi, C. Zhou, R. Biolo, E. Menegaldo, G. Jori

**Affiliations:** Department of Biology, University of Padova, Italy.

## Abstract

The pharmacokinetics of Zn-phthalocyanine (Zn-Pc) in mice bearing a transplanted MS-2 fibrosarcoma has been studied using dipalmitoyl-phosphatidylcholine (DPPC) liposomes and low density lipoproteins (LDL) as drug delivery systems. LDL induce a higher Zn-Pc uptake by the tumour and improve the selectivity of tumour targeting as compared to DPPC liposomes. Experimental photodynamic therapy (PDT) of the MS-2 fibrosarcoma has been performed using liposome-delivered Zn-Pc and the efficiency of tumour necrosis has been measured following four different irradiation protocols. We found that Zn-Pc doses as low as 0.07-0.35 mg kg-1 are sufficient for inducing an efficient tumour response that is linearly dependent on the injected dose. The amount of Zn-Pc in the tumour decreases very slowly as a function of time, hence PDT gives satisfactory results even if performed at relatively long time intervals after administration.


Br. J. Cancer (1990), 61, 407-411 © Macmillan Press Ltd., 1990

### Liposome- or LDL-administered Zn (II)-phthalocyanine as a photodynamic agent for tumours. I. Pharmacokinetic properties and phototherapeutic efficiency

E. Reddi[1], C. Zhou[2], R. Biolo[1], E. Menegaldo[1] & G. Jori[1]

[1]*Department of Biology, University of Padova, via Loredan 10, I-35131 Padova, Italy; and* [2]*Cancer Institute, Chinese Academy of Medical Science, 100021 Beijing, China.*

**Summary** The pharmacokinetics of Zn-phthalocyanine (Zn-Pc) in mice bearing a transplanted MS-2 fibrosarcoma has been studied using dipalmitoyl-phosphatidylcholine (DPPC) liposomes and low density lipoproteins (LDL) as drug delivery systems. LDL induce a higher Zn-Pc uptake by the tumour and improve the selectivity of tumour targeting as compared to DPPC liposomes. Experimental photodynamic therapy (PDT) of the MS-2 fibrosarcoma has been performed using liposome-delivered Zn-Pc and the efficiency of tumour necrosis has been measured following four different irradiation protocols. We found that Zn-Pc doses as low as 0.07–0.35 mg $kg^{-1}$ are sufficient for inducing an efficient tumour response that is linearly dependent on the injected dose. The amount of Zn-Pc in the tumour decreases very slowly as a function of time, hence PDT gives satisfactory results even if performed at relatively long time intervals after administration.

PDT is a modality for the treatment of solid tumours which is based on the use of tumour-localising photosensitisers and irradiation of the tumour with visible light. At the clinical level, this technique is most frequently applied using haematoporphyrin or its derivatives (HpD, Photofrin II) as phototherapeutic agents with red light and satisfactory results have been generally reported (Dougherty, 1984; Hayata *et al.*, 1982; Shumaker & Hetzel, 1987). However, there is now general agreement that these porphyrins are not ideal photosensitisers owing to the small efficiency of red light absorption and chemical heterogeneity (Kessel, 1986*a*). Other photosensitisers, such as phthalocyanines, are being studied *in vitro* and *in vivo* as possible candidates for replacing HpD in PDT (Spikes, 1986). The phthalocyanines are characterised by strong light absorption in the 670–690 nm region ($\varepsilon \simeq 10^5$ $M^{-1}cm^{-1}$) and can efficiently photosensitise both the oxidation of simple substrates (Langlois *et al.*, 1986) and the killing of cultured cells (Ben-Hur & Rosenthal, 1985; Chan *et al.*, 1986). Moreover, phthalocyanines are accumulated and retained by experimental tumours, especially those of the central nervous system (Rosseau *et al.*, 1985; Tralau *et al.*, 1987*a*) causing their destruction upon illumination with red light (Brasseur *et al.*, 1987; Tralau *et al.*, 1987*b*). Most investigations have been performed with the sulphonated derivatives of the phthalocyanines because of their high water solubility. The chemical procedure used in obtaining the sulphonated phthalocyanines generally gives a mixture of molecules with a different degree of sulphonation, while the non-sulphonated phthalocyanines can be easily obtained with a very high purity (Moser & Thomas, 1983). The latter are very hydrophobic compounds and can be used *in vivo* only after their inclusion into lipid matrices. We have reported a procedure for the incorporation of Zn-Pc into small unilamellar vesicles of DPPC (Valduga *et al.*, 1987). The photosensitising efficiency of this phthalocyanine *in vitro* has also been documented (Valduga *et al.*, 1988).

We have previously shown that serum lipoproteins are the exclusive carriers of Zn-Pc '*in vivo*' (Reddi *et al.*, 1987). The transport of photosensitising (Reddi *et al.*, 1987) and cytostatic (Vitols *et al.*, 1985) agents by LDL has been proposed in order to improve the selectivity of tumour targeting. Photofrin II is also delivered to cultured fibroblasts by LDL via a receptor-mediated endocytotic mechanism; this porphyrin is taken up by cells more efficiently when complexed with LDL than other serum proteins (Candide *et al.*, 1986).

Correspondence: G. Jori.
Received 27 July 1989; and in revised form 31 October 1989.

In this paper we report a comparative pharmacokinetic study of Zn-Pc injected into tumour-bearing mice after incorporation into DPPC liposomes (Zn-Pc-DPPC) or *in vitro* complexation to human LDL (Zn-Pc-LDL). PDT has been performed after the administration of Zn-Pc-DPPC and the extent of the photoinduced tumour necrosis has been measured as a function of different experimental parameters.

### Materials and methods

### *Chemicals*

Zn-Pc was a gift from Ciba-Geigy (Basel, Switzerland) and has been used as received. Previous analysis of the compound showed a degree of purity of 97% (Valduga *et al.*, 1987). DL-α-DPPC was a product of Sigma Chemical Co. All other chemicals and solvents were analytical grade reagents.

### *Animals and tumour*

Female Balb/c mice (18–20 g body weight) obtained from Charles River (Como, Italy) have been used as experimental model. The mice were grown in cages with free access to standard dietary chow and tap water. Animal care was made according to the guidelines established by the Italian committee for experiments on animals. The MS-2 fibrosarcoma has been supplied by Istituto Nazionale dei Tumori, Milan. The tumour was implanted in the right hind leg of the mice by injection of 0.25 ml of a cell suspension containing at least $10^6$ cells $ml^{-1}$. All the experiments were started at 8 days after tumour implantation, when its diameter was 0.7–0.8 cm. When necessary, the mice were anaesthetised by i.p. injection of ketalar (150 mg $kg^{-1}$).

### *Drug preparation*

Zn-Pc was incorporated into small unilamellar vesicles of DPPC following the procedure described by Valduga *et al.* (1987). For these experiments liposomes were prepared in 0.9% aqueous NaCl and before injection they were dialysed for 3 h against 250 ml of NaCl with a change of the NaCl solution after the first hour.

The Zn-Pc was complexed *in vitro* with human LDL after isolation of this lipoprotein fraction ($1.006 < d < 1.063$) by density gradient ultracentrifugation (Havel *et al.*, 1955) of blood samples obtained from normolipidaemic volunteers. The isolated LDLs were dialysed overnight at 4°C, against 0.01 M phosphate buffer, pH 7.4, with a change of the buffer after the first hour. The Zn-Pc was incorporated into LDL by the slow addition of a 1 mM stock solution of the drug in pyridine, which had been 5-fold diluted with absolute ethanol, to an aqueous solution, pH 7.4, of LDL($\simeq$7 mg ml$^{-1}$). The added volume of the Zn-Pc solution was one-tenth the volume of the lipoprotein solution. During the addition, the lipoprotein solution was kept at 37°C. In order to remove the organic solvents, the Zn-Pc-LDL complex was dialysed at room temperature for 3 h against phosphate buffer at pH 7.4 with one change of the buffer after the first hour.

The Zn-Pc concentration in the liposomal vesicles or in the lipoproteins was measured by absorbence at 673 nm using $\varepsilon = 2.41 \times 10^5$ M$^{-1}$cm$^{-1}$ in pyridine (value determined in our laboratory).

*Pharmacokinetic studies*

The Zn-Pc-DPPC or the Zn-Pc-LDL were injected i.v. into tumour-bearing mice at a dose of 0.12 mg kg$^{-1}$. At different times after injection the animals were killed, serum samples as well as the tumour and several normal tissues were collected for the analysis of their Zn-Pc content. The tissues were washed with saline solution and frozen until the analysis was performed. The concentration of Zn-Pc in the serum and in the tissues was measured spectrophotofluorimetrically following the procedure previously described by Reddi *et al.* (1987). The assay method was essentially based on tissue homogenisation in 2% SDS and measurement of the fluorescence emission intensity of Zn-Pc in the supernatant was obtained after centrifugation of the homogenate. This procedure allows an essentially quantitative extraction of the drug from tissues (Reddi *et al.*, 1987).

*Photodynamic therapy*

The experimental PDT with Zn-Pc-DPPC was performed by irradiation of the tumour area with red light (590–750 nm) isolated from a 250 W halogen lamp equipped with a set of optical filters. The emitted light was focused into a bundle of optical fibres having a total diameter of 0.6 cm. The fibre tip was placed at 1 cm from the tumour surface during the phototreatments. Tumour irradiation was performed according to different protocols (Table I) in order to examine the effect of various experimental parameters on the extent of the photoinduced tumour necrosis.

The estimation of the extent of tumour necrosis was performed at 24 h after PDT. The procedure adopted involved the fixation of the tumour in 4% formalin, followed by sectioning of the tumour at 2 mm intervals. The width and depth of the necrotic area were measured for each tissue slice. The maximum values of width and depth were recorded for each tumour and their product was chosen for a quantitative evaluation of the tumour response. Each point, reported in the figures describing the PDT data, represents the mean (± s.d.) from at least three animals.

### Results

*Pharmacokinetic studies*

Serum samples taken from mice at various times after i.v. injection of Zn-Pc were analysed by column chromatography as previously described (Reddi *et al.*, 1987). Both DPPC- and LDL-administered Zn-Pc were found to be exclusively associated with the lipoprotein fraction, similar to what we have observed for i.p.-injected Zn-Pc-DPPC (Reddi *et al.*, 1987). The elimination of Zn-Pc from the serum follows a biphasic kinetics. About 70% of the drug is eliminated in about 12 h while the remaining fraction is eliminated rather slowly: Zn-Pc levels as low as approx. 40 ng ml$^{-1}$ are present in the serum at 7 days after the i.v. injection. The kinetics of Zn-Pc clearance from the serum are independent of the modality used for administration, i.e. via liposomes or LDL.

In Figures 1 and 2 we show the time-dependence of Zn-Pc concentration in selected tissues after i.v. administration of Zn-Pc-DPPC and Zn-Pc-LDL, respectively. The recoveries were estimated for the tumour, the muscle, i.e. the tissue where the tumour is implanted, and the liver, i.e. the normal tissue which usually shows a large uptake of systemically injected photosensitisers (Dougherty, 1984). Each experimental point represents the average of at least three independent determinations performed on specimens obtained from different animals. In the case of Zn-Pc-DPPC the maximum concentration of drug in the tumour was reached by 3 h after administration (Figure 1), while for the Zn-Pc-LDL about a two-fold greater maximum accumulation is observed at about 24 h (Figure 2). On the other hand, rather similar recoveries of Zn-Pc from the liver and muscle are obtained upon injection of Zn-Pc-DPPC and Zn-Pc-LDL.

The degree of selectivity of drug localisation in the tumour is usually expressed by the ratio of its concentration in the tumour and selected normal tissues. In Table II we report the ratios tumour/liver and tumour/muscle for Zn-Pc at different times after injection.

**Figure 1** Time dependence of Zn-Pc concentration in tumour (●——●), liver (○——○) and muscle (△——△) of mice injected with 0.12 mg kg$^{-1}$ Zn-Pc-DPPC.

**Figure 2** Time dependence of Zn-Pc concentration in tumour (●——●), liver (○——○) and muscle (△——△) of mice injected with 0.12 mg kg$^{-1}$ Zn-Pc-LDL.

**Table I** Protocols used in the PDT of the MS-2 fibrosarcoma

| *Protocol* | *Injected Zn-Pc dose (mg kg$^{-1}$)* | *Irradiation dose rate (mW cm$^{-2}$)* | *Total light dose (J cm$^{-2}$)* | *Δt (h)* |
|---|---|---|---|---|
| 1 | 0.14 | 50–200 | 300 | 24 |
| 2 | 0.14 | 180 | 150–450 | 24 |
| 3 | 0.14 | 180 | 300 | 3–72 |
| 4 | 0.07–0.35 | 180 | 300 | 24 |

Δt is the time interval between the Zn-Pc injection and PDT.

**Table II** Ratio between the concentration of Zn-Pc in the tumour and liver and in the tumour and muscle for Zn-Pc-DPPC and Zn-Pc-LDL

| | *Tumour/liver* | | *Tumour/muscle* | |
|---|---|---|---|---|
| *Time (h)* | *Zn-Pc-DPPC* | *Zn-Pc-LDL* | *Zn-Pc-DPPC* | *Zn-Pc-LDL* |
| 3 | 0.45 | 0.47 | 2.82 | 2.89 |
| 12 | 0.70 | 1.25 | 3.30 | 4.37 |
| 24 | 0.94 | 1.85 | 3.71 | 5.71 |
| 48 | 1.01 | 1.32 | 2.80 | 4.95 |
| 168 | 1.04 | 2.27 | 3.84 | 4.20 |

*Experimental photodynamic therapy*

Generally, the tumours irradiated with red light after the i.v. injection of Zn-Pc-DPPC showed the appearance of a dark spot within a few hours, which gradually evolved into eschar formation and loss of part of the tumour mass. In all the experiments reported here, the extent of tumour response, as a function of different parameters, has been evaluated at 24 h after irradiation, i.e. before the loss of any tumour mass.

In Figure 3 we show the extent of the tumour necrosis as a function of the irradiation dose rate (Table I, protocol 1). Clearly, the tumour necrosis increases upon increasing the dose rate. With our light source, tumour necrosis was observed also in mice not injected with Zn-Pc but only upon irradiation with dose rates higher than 230 mW cm$^{-2}$, possibly owing to the onset of thermal damage. The extent of the necrotic area depends also on the total light dose as shown by the data obtained with mice irradiated following protocol 2 (Figure 4). It appears that, under our conditions, only light doses above 150 J cm$^{-2}$ induce detectable tumour necrosis. For a fixed dose rate and total light dose (protocol 3), the tumour necrosis is dependent on the time interval between drug administration and PDT (Figure 5). Significant tumour necrosis is observed for irradiation performed up to 70 h after the Zn-Pc injection, which is in agreement with the slow clearance of Zn-Pc from the tumour demonstrated by our pharmacokinetic studies. PDT was not performed at time intervals longer than 70 h after Zn-Pc injection, because of the too large tumour dimensions and the appearance of spontaneous necrosis. It is also clear from Figure 6 that a linear relationship exists between the extent of the photoinduced necrotic area and the injected dose of photosensitiser. Such a behaviour is not surprising owing to the low Zn-Pc doses used in our experiments. The upper limit of the photosensitiser dose tested by us was 0.35 mg kg$^{-1}$, since at this dose the photoinduced necrosis involved the whole tumour mass.

**Figure 3** Extent of the photoinduced tumour necrosis as a function of the light dose rate. The mice have been treated according to protocol 1.

**Figure 4** Extent of the tumour necrosis as a function of the total light dose. The irradiation conditions are those of protocol 2.

**Figure 5** Extent of the tumour necrosis as a function of the time interval between Zn-Pc administration and irradiation. For experimental conditions see protocol 3.

**Figure 6** Extent of the photoinduced tumour necrosis as a function of the injected Zn-Pc dose. Experimental conditions as in protocol 4.

### Discussion

Our data show that relatively large Zn-Pc concentrations can be complexed '*in vitro*' with isolated LDL; thus, this lipoprotein class can be used '*in vivo*' as a Zn-Pc delivery system. The occurrence of an active LDL-receptor pathway for the delivery of Zn-Pc to our tumour model is suggested by the about two-fold larger concentrations of phthalocyanine observed in the tumour after injection of the Zn-Pc-LDL as compared with Zn-Pc-DPPC. However, even with LDL as a carrier, the selectivity of tumour targeting by Zn-Pc is limited by the fact that LDL-bound Zn-Pc once injected undergoes a partial redistribution among other lipoprotein classes, mainly high density lipoproteins (data not shown). The actual mechanism of Zn-Pc delivery from LDL (and/or other carriers) to the tumour tissue is certainly complex and probably involves the time-dependent redistribution of the dye among different tissue compartments. A similar behaviour has been observed for sulphonated porphine derivatives and HpD components (Kessel *et al.*, 1987; Kessel, 1986*b*). This might explain the lower efficiency of PDT at 3 h as compared to 24 h after Zn-Pc injection (Figure 5) in spite of the almost identical overall concentration of the photosensitiser in the tumour (Figure 1).

In any case, it is likely that Zn-Pc, once released inside the tumour cell, becomes associated with apolar compartments owing to its hydrophobic character. Ultrastructural studies on mouse tumour tissues, which had been irradiated in the presence of Zn-Pc, showed that the cytoplasmic and mitochondrial membranes were heavily damaged (Milanesi *et al.*, 1987). This endocellular distribution of Zn-Pc can explain the slow release of this drug from the tumour. We have previously demonstrated that Zn-Pc is eliminated from the body by the bile–gut pathway (Reddi *et al.*, 1987). This requires the removal of Zn-Pc from the tumour and its transport to the liver by serum proteins; thus the slow clearance of Zn-Pc may depend on the low accessibility of the drug binding sites to the protein carriers. The poor lymphatic drainage typical of tumour tissues has also been invoked to explain the slow release of photosensitising drugs (Bugelski *et al.*, 1981). The high degree of selectivity of tumour targeting by our procedure for Zn-Pc administration and transport is underlined by the fact that at 24 h after injection similar or larger amounts of Zn-Pc are found in the tumour as compared to the liver (see Figures 1 and 2). Moreover, the constantly low levels of Zn-Pc in the muscle should indicate that there is only a minimal risk of damage to normal tissues adjacent to the tumour during PDT.

The slow clearance of Zn-Pc by the tumour also suggests the possibility of multiple phototreatments following a single Zn-Pc injection. Our PDT data show that irradiations performed at 70 h after Zn-Pc administration induce an efficient tumour necrosis and, on the basis of our pharmacokinetic data (Figure 1), it is likely that similar tumour response are obtained by PDT treatments performed at 7 days after Zn-Pc injection. These considerations are reinforced by the finding (Figure 6) that Zn-Pc doses as low as $0.07\ \mathrm{mg\,kg^{-1}}$ are sufficient for inducing an important tumour necrosis. The linearity of the plot of the tumour responses against Zn-Pc dose (Figure 6) is due to the low amounts of photosensitising agent used in our experiments. On the other hand, Tralau *et al.* (1987*b*) found that the extent of tumour response to PDT tends toward a plateau value upon injection of sulphonated Al-Pc doses above $1\ \mathrm{mg\,kg^{-1}}$. It is likely that in the presence of large dye concentrations in the tumour the incident light is efficiently absorbed only by the superficial layers of the tissue, thus reducing the optical penetration depth.

The extent of the tumour necrosis for a given Zn-Pc dose depends on the total light dose (see Figure 4), as already observed by other authors for porphyrins (Fingar *et al.*, 1987) and phthalocyanines (Tralau *et al.*, 1987*b*) and expected on the basis of theoretical considerations (Doiron *et al.*, 1984). The extent of tumour necrosis depends also on the irradiation dose rate (see Figure 3). In particular, we observed about a 6-fold increase of the necrotic area from 50 to $200\ \mathrm{mW\,cm^{-2}}$. For unsensitised animals no tumour necrosis could be detected up to $230\ \mathrm{mW\,cm^{-2}}$. Moreover, upon irradiation of Zn-Pc-injected mice with dose rates around $180\ \mathrm{mW\,cm^{-2}}$, the temperature increase of the tumour tissue, as measured with an infra red sensitive telecamera (Vietri *et al.*, 1988), was below 3–4°C, which is lower than that usually considered to originate hyperthermal effects (Evensen & Moan, 1988). However, a synergism between this modest temperature increase and photodynamic effects cannot be ruled out. The occurrence of such a synergism might explain the upward deviation from linearity observed above about $130\ \mathrm{mW\,cm^{-2}}$ in the plot describing the influence of dose rate on the extent of the necrotic area (Figure 3).

Our pharmacokinetic studies show that a fraction of the Zn-Pc is cleared from the serum at a slow rate; this circumstance may be responsible for some cutaneous photosensitivity (Zalar *et al.*, 1977). However, our pharmacokinetic studies showed that only negligible amounts of Zn-Pc (around $0.1\ \mu\mathrm{g\,g^{-1}}$ tissue) are accumulated in the skin between 3 h and 1 week after administration. This should minimise the risk of skin photosensitisation. The latter should be also minimised by the spectral properties typical of phthalocyanines which absorb ambient light much less efficiently than porphyrins.

In conclusion Zn-Pc appears to be a very promising photodynamic agent for the therapy of tumour due to its selective localisation in and slow clearance from the tumour, and the efficient photosensitisation of tumour necrosis even upon injection at very low doses.

This work received financial support from Consiglio Nazionale delle Ricerche, Italy, under the special project 'Oncologia', contract no. 87.01322.44.

### References

BEN-HUR, E. & ROSENTHAL, I. (1985). The phthalocyanines: a new class of mammalian cells photosensitizers with a potential for cancer phototherapy. *Int. J. Radiat. Biol.*, **47**, 145.

BRASSEUR, N., ALI, H., LANGLOIS, R., WAGNER, J.R., ROUSSEAU, J. & VAN LIER, J.E. (1987). Biological activities of phthalocyanines. V. Photodynamic therapy of EMT-6 mammary tumors in mice with sulfonated phthalocyanines. *Photochem. Photobiol.*, **45**, 581.

BVGELSKI, P.J., PORTER, C.W. & DOUGHERTY, T.J. (1981). Autoradiographic distribution of HpD in normal and tumour tissue of the mouse. *Cancer Res.*, **41**, 4606.

CANDIDE, C., MORLIERE, P., MAZIERE, J.C. & 5 others (1986). *In vitro* interaction of the photoactive anticancer porphyrin derivative photofrin II with low density lipoprotein, and its delivery to cultured human fibroblasts. *FEBS Lett.*, **207**, 133.

CHAN, W.S., SVENSEN, R., PHILLIPS, D. & HART, I.R. (1986). Cell uptake, distribution and response to aluminum chlorosulphonated phthalocyanine, a potential antitumour photosensitiser. *Br. J. Cancer*, **53**, 255.

DOIRON, D.R., GOMER, C.J., FOUNTAIN, S.W. & RAZUM, N.J. (1984). Photophysics and dosimetry of photoradiation therapy. In *Porphyrins in Tumor Phototherapy*, Andreoni, A. & Cubeddu, R. (eds) p. 281. Plenum Press: New York.

DOUGHERTY, T.J. (1984). Photodynamic therapy (PDT) of malignant tumors. *CRC Rev. Oncol. Hematol.*, **2**, 83.

EVENSEN, J.F. & MOAN, J. (1988). Photodynamic therapy of C3H tumours in mice: effect of drug/light dose fractionation and misonidazole. *Lasers Med. Sci.*, **3**, 1.

FINGAR, V.H., POTTER, W.R. & HENDERSON, B.W. (1987). Drug and light dose dependence of photodynamic therapy: a study of tumor cell clonogenicity and histologic changes. *Photochem. Photobiol.*, **45**, 643.

HAVEL, R.J., EDER, H.A. & BRAGDON, J.H. (1955). Distribution and chemical composition of ultracentrifugally separated lipoproteins in human serum. *J. Clin. Invest.*, **34**, 1345.

HAYATA, Y., KONAKA, C., TAKIZAWA, N. & KATO, H. (1982). Hematoporphyrin derivative and laser photoradiation in the treatment of lung cancer. *Chest*, **81**, 269.

KESSEL, D. (1986*a*). Proposed structure of the tumor-localizing fraction of HpD. *Photochem. Photobiol.*, **44**, 193.

KESSEL, D. (1986*b*). Sites of photosensitization by derivatives of hematoporphyrin. *Photochem. Photobiol.*, **44**, 489.

KESSEL, D., THOMPSON, P., SOATIO, K. & NANTURI, K.D. (1987). Tumor localization and photosensitization by sulfonated derivatives of tetraphenylporphine. *Photochem. Photobiol.*, **45**, 787.

LANGLOIS, R., ALI, H., BRASSEUR, N., WAGNER, J.R. & VAN LIER, J.E. (1986). Biological activities of phthalocyanines. IV. Type II sensitized photooxidation of L-tryptophan and cholesterol by sulfonated metallo phthalocyanines. *Photochem. Photobiol.*, **44**, 117.

MILANESI, C., BIOLO, R., REDDI, E. & JORI, G. (1987). Ultrastructural studies on the mechanism of the photodynamic therapy of tumors. *Photochem. Photobiol.*, 46, 675.

MOSER, F.H.& THOMAS, A.L. (1983). *The Phthalocyanines, vols I and II*. CRC Press: Boca Raton.

REDDI, E., LO CASTRO, G., BIOLO, R. & JORI, G. (1987). Pharmacokinetic studies with zinc(II)-phthalocyanine in tumour-bearing mice. *Br. J. Cancer*, **56**, 597.

ROSSEAU, J., ALI, H., LAMOUREUX, G., LEBEL, J.E. & VAN LIER, J.E. (1985). Synthesis, tissue distribution and tumor uptake of $^{99m}$Tc- and $^{67}$Ga-tetrasulfophthalocyanine. *Int. J. Appl. Radiat. Isot.*, **36**, 709.

SHUMAKER, B.P. & HETZEL, F.W. (1987). Clinical laser photodynamic therapy in the treatment of bladder carcinoma. *Photochem. Photobiol.*, **46**, 899.

SPIKES, J.D. (1986). Phthalocyanines as photosensitizers in biological systems and for the photodynamic therapy of tumors. *Photochem. Photobiol.*, **43**, 691.

TRALAU, C.J., BARR, H., SANDEMAN, D.R., BARTON, T., LEWIN, M.R. & BOWN, S.G. (1987*a*). Aluminum sulfonated phthalocyanine. Distribution in rodent tumors of the colon, brain and pancreas. *Photochem. Photobiol.*, **46**, 777.

TRALAU, C.J., MACROBERT, A.J., COLERIDGE-SMITH, P.D., BARR, H. & BOWN, S.G. (1987*b*). Photodynamic therapy with phthalocyanine sensitisation: quantitative studies in a transplantable rat fibrosarcoma. *Br. J. Cancer*, **55**, 389.

VALDUGA, G., NONELL, S., REDDI, E., JORI, G. & BRASLAVSKY, S.E. (1988). The production of singlet molecular oxygen by zinc (II)phthalocyanine in ethanol and in unilamellar vesicles. Chemical quenching and phosphorescence studies. *Photochem. Photobiol..*, **48**, 1.

VALDUGA, G., REDDI, E. & JORI, G. (1987). Spectroscopic studies on Zn(II)-phthalocyanine in homogenous and microheterogeneous systems. *J. Inorg. Biochem.*, **25**, 59.

VIETRI, F., GIROLAMI, M., JORI, G., BIOLO, R., REDDI, E. & SALCITO, G. (1988). Zinc-phthalocyanine as a phototherapeutic and photodiagnostic agent for tumours. Abstracts International Conference on Photodynamic Therapy, London, Abstract No. 130.

VITOLS, S.G., MASQUELIER, M. & PETERSON, C.O. (1985). Selective uptake of a toxic lipophilic anthracycline derivative by low-density lipoprotein receptor pathway in cultured fibroblasts. *J. Med. Chem.*, **28**, 451.

ZALAR, G.L., POH-FITZPATRICK, M., KROHN, D.L., JACOBS, R. & HARBER, L.C. (1977). Induction of drug photosensitization in man after parenteral exposure to hematoporphyrin. *Arch. Dermatol.*, **113**, 1392.